# SLC26A9 in triple-negative breast cancer stem cells: a network pharmacology and molecular modeling study

**DOI:** 10.3389/fbioe.2025.1703343

**Published:** 2025-11-13

**Authors:** Mimi Shen, Zhiyuan Ma, Yanghui Cao, Lan Wang, Guoli Feng, Zhengxing Zhou, Leilei Li, Bei Ji, Shuhui Liu, Jiaqi Qin, Qin Wang, Xuemei Liu, Taolang Li

**Affiliations:** 1 Department of General Surgery, Affiliated Hospital of Zunyi Medical University, Zunyi, China; 2 Department of Thyroid and Breast Surgery, Affiliated Hospital of Zunyi Medical University, Zunyi, China; 3 Department of Gastroenterology, Digestive Disease Hospital, Affiliated Hospital of Zunyi Medical University, Zunyi, China

**Keywords:** triple-negative breast cancer, cancer stem cells, SLC26A9, systems pharmacology, network pharmacology, molecular modeling, TP53, precision oncology

## Abstract

Triple-negative breast cancer (TNBC) presents significant clinical challenges due to its high heterogeneity and lack of effective targeted therapies. Cancer stem cells (CSCs) play a crucial role in TNBC recurrence, metastasis, and drug resistance. However, the interplay between ion transport, microenvironmental regulation, and classical stemness pathways remains underexplored in existing reviews. In this work, we systematically integrated multi-omics databases, network pharmacology, protein–protein interaction (PPI) analysis, functional pathway enrichment, and molecular modeling to highlight the “bridging” role of SLC26A9 and its interacting proteins in TNBC stem cell self-renewal, drug resistance, and microenvironmental regulation. Comprehensive molecular docking and 100-ns molecular dynamics (MD) simulations demonstrated that the small molecule S9-A13 exhibited high affinity and stable binding to both SLC26A9 and tumor protein p53 (TP53), with docking affinities of −7.737 and −8.447 kcal/mol and molecular mechanics/generalized Born surface area (MM/GBSA) binding free energies of −34.47 and −25.65 kcal/mol, respectively. These results suggest that S9-A13 may act on the SLC26A9–TP53 axis to enable multi-target regulation of TNBC cancer stem cells. We further discuss the translational implications of such interventions, including safety profile considerations, potential off-target effects, and delivery strategies. In summary, this review provides a structured framework and testable hypotheses for developing SLC26A9-based multi-target precision therapies for TNBC CSCs, while emphasizing that these computational findings are hypothesis-generating and require rigorous experimental and clinical validation prior to translation.

## 1 Introduction

Triple-negative breast cancer (TNBC) is the most aggressive and heterogeneous subtype of breast cancer, characterized by limited therapeutic options and a poor prognosis (Bai et al., 2021; Zagami and Carey, 2022). Due to the absence of estrogen receptor (ER), progesterone receptor (PR), and human epidermal growth factor receptor 2 (HER2) expression, conventional targeted therapies are largely ineffective for TNBC patients, who remain reliant on chemotherapy and immunotherapy (Mas et al., 2020; Ye et al., 2023). However, the presence of highly heterogeneous tumor cell subpopulations—particularly cancer stem cells (CSCs) with self-renewal and drug resistance capabilities—drives tumor recurrence, metastasis, and therapeutic resistance (Hua et al., 2022; Zeng et al., 2023; Saw et al., 2024). The persistence of CSCs, tightly coupled to intricate signaling and microenvironmental cues, remains a major barrier to precision therapy.

Recent advances in systems pharmacology, network pharmacology, and multi-omics integration have facilitated the discovery of tumor targets and drug development (Dong et al., 2024; Hu et al., 2023). Nevertheless, effective multi-target precision interventions for TNBC CSCs remain elusive. Recent studies have demonstrated that calcium ion channels play critical roles in the regulation of breast cancer stem cells (BCSCs), influencing their self-renewal, proliferation, and metastatic potential (Zhuo et al., 2024; Mukhopad et al., 2023). However, anion transporters such as chloride/bicarbonate exchangers remain insufficiently explored, underscoring the novelty of investigating SLC26A9 in the context of BCSC biology.

Traditional single-target drugs often exhibit limited efficacy due to signaling pathway compensation and tumor microenvironment adaptation. Ion channel proteins, especially SLC26A9—a novel transmembrane transporter—have been shown to play unique roles not only in intracellular ion homeostasis, polarity maintenance, and microenvironmental pH regulation, but also in the biology of solid tumors and CSCs (Chang et al., 2009; Liu et al., 2022; Zhang et al., 2024).

In this review, we focus on the continuum: “TNBC CSCs → signaling networks → ion channels → multi-target drug development.” We integrate multi-omics data, protein–protein interaction (PPI) and enrichment analyses, and molecular modeling to propose a systems pharmacology perspective on SLC26A9 and its interactome in regulating TNBC CSCs. Additionally, we explore the multi-target potential of S9-A13 on the SLC26A9–TP53 axis as hypothesis-generating evidence. Finally, we outline translational opportunities and challenges, providing a conceptual framework and testable hypotheses for future validation.

## 2 Triple-negative breast cancer stem cells (TNBC CSCs): the core barrier of heterogeneity and precision therapy

### 2.1 Molecular and biological characteristics of TNBC CSCs

Triple-negative breast cancer (TNBC) is the most heterogeneous and aggressive subtype of breast cancer. Its biological foundation lies in the presence of a small subpopulation of cells with stem-like properties, known as cancer stem cells (CSCs) (Hua et al., 2022). These CSCs exhibit robust self-renewal, differentiation, and regenerative capacities, continuously reconstituting the tumor’s diversity and adaptability.

Studies have shown that TNBC CSCs are typically identified by surface markers such as CD44+/CD24− and ALDH+, and both their abundance and functional activity are significantly higher than those observed in other breast cancer subtypes (Dionísio et al., 2020; Murthy et al., 2024). These CSC subpopulations confer strong invasive and drug-resistant properties upon TNBC, acting as the “root system” driving tumor initiation, recurrence, metastasis, and therapeutic resistance (Zhang et al., 2023; Wang L. et al., 2024; Liu et al., 2024).

Notably, CSCs display inherent resistance to chemotherapy and remarkable adaptability to the tumor microenvironment, making the eradication of these “seed” cells particularly challenging with conventional therapies. This represents a major obstacle in clinical management of TNBC (Dakal et al., 2024; Li et al., 2021). Collectively, these features argue for regulators that link stemness maintenance to tumor microenvironment (TME) adaptation as priority targets for intervention.

### 2.2 Complex signaling pathways and microenvironmental regulation

The fate and maintenance of CSCs are highly dependent on intricate, multi-pathway, and multi-level signaling networks (Clara et al., 2020; Yang et al., 2020). Classical stem cell signaling pathways—including Notch, Wnt/β-catenin, phosphoinositide 3-kinase (PI3K)/protein kinase B (AKT), and Janus kinase (JAK)/signal transducer and activator of transcription (STAT)—form the core network governing CSC self-renewal, proliferation, and differentiation (Dakal et al., 2024; Yang et al., 2020). Additionally, mutations or inactivation of tumor suppressor genes such as TP53 enable TNBC CSCs to evade immune surveillance and apoptosis, thereby enhancing their malignant phenotype and survival capacity (Wang Z. et al., 2024).

Recent evidence indicates that metabolic reprogramming, ion channel dysfunction, and dynamic changes in the tumor microenvironment (TME)—including acidification, hypoxia, and immune suppression—also play critical roles in the survival and drug resistance of CSCs (Dakal et al., 2024; Chen et al., 2021; El-Sahli and Wang, 2020; O'Reilly and Buchanan, 2019). The interplay of these multidimensional signals, genetic alterations, and environmental factors contributes to the highly complex and dynamically adaptable regulatory network of CSCs. This network complexity is a fundamental reason why traditional “single-pathway targeted therapies” have failed to achieve significant clinical breakthroughs. The multilayered cross-talk among these factors helps explain why single-pathway targeting has yielded only limited and transient benefits in TNBC.

### 2.3 Bottlenecks in precision therapy and opportunities for systems pharmacology

Although the cancer stem cell (CSC) theory has stimulated the development of several novel anticancer agents, there have been no substantial clinical breakthroughs targeting TNBC CSCs. The central challenge lies in accurately identifying key regulatory nodes that govern CSC survival and drug resistance, as well as discovering novel targets that enable multi-target, cross-pathway synergistic interventions. In recent years, emerging research paradigms such as systems pharmacology, network pharmacology, and multi-omics integration have provided both theoretical foundations and methodological tools for multi-target interventions within CSC networks.

In summary, the high complexity and dynamic plasticity of CSC regulatory networks fundamentally limit the efficacy of existing single-target therapies and contribute to the frequent emergence of drug resistance. Therefore, systems pharmacology and multi-target interventions represent promising new avenues to overcome these current bottlenecks. Within this context, ion-transport modulators—exemplified by SLC26A9—warrant focused evaluation as potential “bridge” targets for enabling cross-pathway, multi-target therapeutic strategies.

## 3 SLC26A9: a novel role for transmembrane ion channel proteins in cancer stem cell regulation

### 3.1 Molecular structure and functional diversity of SLC26A9

SLC26A9 belongs to the solute carrier 26 (SLC26) family and is an emerging anion transmembrane transporter protein that primarily mediates the transport of ions such as Cl^−^ and HCO_3_^−^ across cellular membranes. It is widely expressed in various epithelial tissues and organs (Zhang et al., 2024; Balázs and Mall, 2018; Kunzelmann et al., 2023). Beyond maintaining intracellular and extracellular ion homeostasis, cellular polarity, and pH balance, SLC26A9 directly participates in signal transduction and adapts to the tumor microenvironment (Calvete et al., 2021; Ma et al., 2025). Compared with other SLC26 family members, such as SLC26A3 and SLC26A4, SLC26A9 exhibits broader tissue distribution and a wider range of biological functions. These characteristics position SLC26A9 to mechanistically influence CSC-relevant processes that are sensitive to ionic and pH dynamics.

### 3.2 Research progress on SLC26A9 in solid tumors and stem cells

A growing body of evidence indicates that SLC26A9 is abnormally expressed in various solid tumors, including gastric, breast, and colorectal cancers. Its dysregulation is closely associated with tumor cell proliferation, migration, apoptosis, and drug resistance (Liu et al., 2022; Zhang et al., 2024; Ma et al., 2025). In the context of cancer stem cells (CSCs), the role of SLC26A9 has attracted increasing attention. As a key regulator of cellular polarity and microenvironmental pH, SLC26A9 may influence CSC self-renewal, differentiation, and survival by modulating intracellular and extracellular ion concentrations and acid–base balance. However, direct mechanistic data in TNBC CSCs remain limited, highlighting the need for hypothesis-driven experimental validation.

### 3.3 SLC26A9: an emerging “bridge target” for multi-target precision therapy

Although mechanistic studies on the role of SLC26A9 in TNBC CSC regulation are still in their early stages, existing research has suggested that SLC26A9 plays important roles in various solid tumors, such as lung cancer. However, its systemic functions in breast cancer stem cells, particularly in triple-negative breast cancer (TNBC) CSCs, remain poorly characterized (Liu et al., 2022; Zhang et al., 2024; Ma et al., 2025). Most current literature focuses on the single physiological functions of SLC26A9 or its involvement in individual signaling pathways. There is a lack of comprehensive analysis regarding its pivotal role as a “bridge” in integrating complex signaling networks, modulating the tumor microenvironment, and coordinating multi-target regulation in stem cells.

Current mainstream therapeutic strategies still predominantly target single molecules or pathways, which are vulnerable to tumor heterogeneity and compensatory signaling effects. This often results in limited clinical efficacy and frequent development of drug resistance. Given the limits of single-pathway strategies, a “bridge” target that coordinates signaling, metabolism, and TME—potentially SLC26A9—may support multi-target precision approaches pending functional confirmation.

## 4 Interaction mechanisms and molecular networks of SLC26A9 in triple-negative breast cancer stem cells

### 4.1 Data sources and reproducibility parameters

To ensure methodological transparency and reproducibility, all databases and analytical tools used in this study were carefully documented with their access dates and key parameters. All searches were restricted to *Homo sapiens*.

1. GeneCards (https://www.genecards.org/, accessed June 21, 2025): The database was queried using the keywords “SLC26A9” and “triple-negative breast cancer stem cells.” All retrieved gene entries were merged after removing duplicates.
2. Venny 2.1 (https://www.bioinformatics.com.cn/, accessed June 21, 2025): The Venny platform was used to identify overlapping targets between the SLC26A9-related and TNBC CSC-related gene sets, resulting in 457 shared genes, which served as the foundation for subsequent analyses.
3. STRING (https://string-db.org/, accessed June 21, 2025): The STRING database was used to construct the protein–protein interaction (PPI) network. Parameters were set as follows: Species: *Homo sapiens*; Confidence score (combined score) > 0.4.
4. Network filtering: Unconnected nodes (degree = 0) were removed to refine the PPI network and improve its reliability.
5. Cytoscape (version 3.9.1): The PPI network was visualized using Cytoscape. Node color intensity was mapped to degree centrality, allowing the identification of key hub proteins.
6. Metascape (https://metascape.org/, accessed June 21, 2025): The Metascape platform was employed for Gene Ontology (GO) functional enrichment and Kyoto Encyclopedia of Genes and Genomes (KEGG) pathway analyses of the 457 shared genes.

### 4.2 Database mining and target identification

**FIGURE 1:**
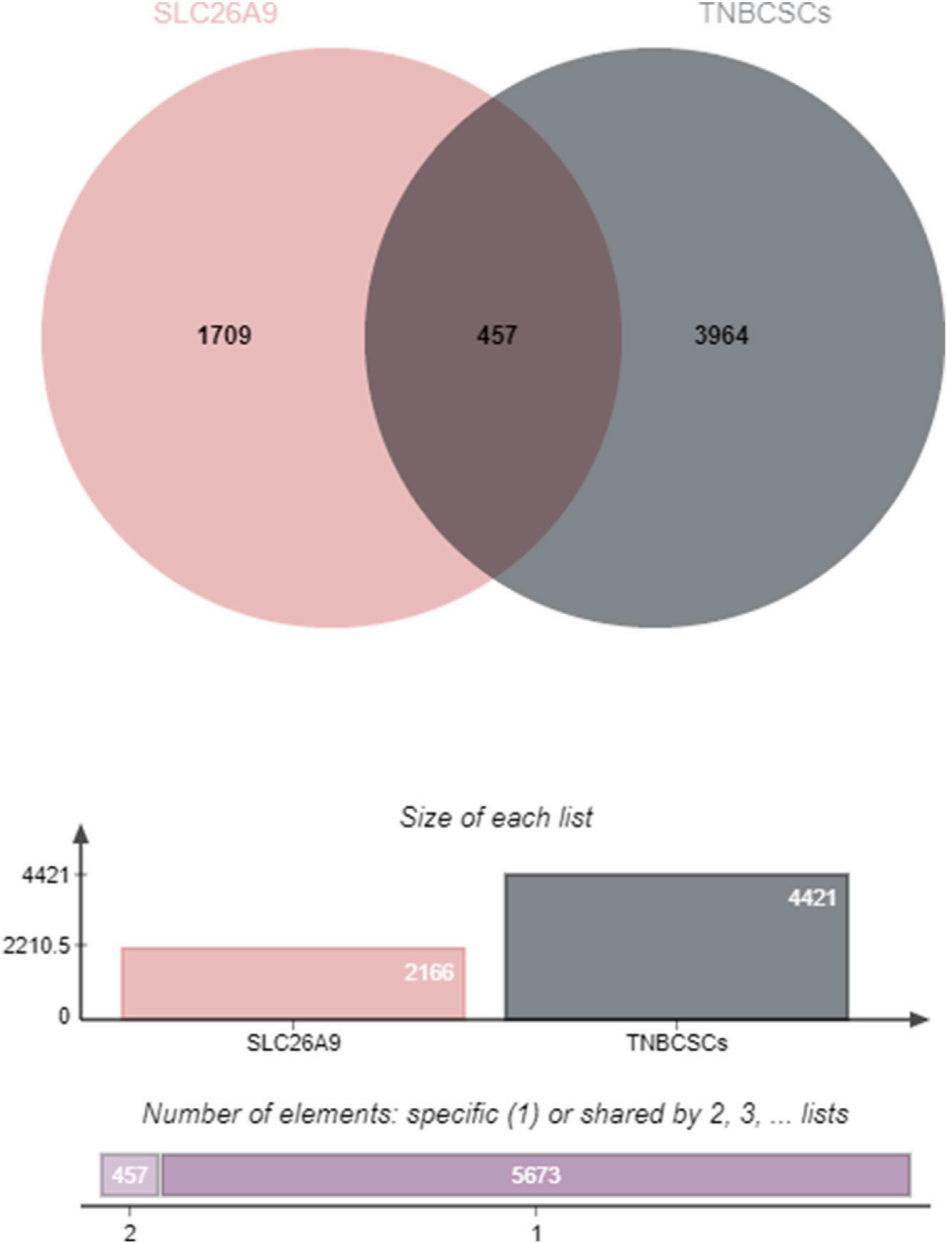
Intersection Analysis of Target Genes Associated with SLC26A9 and Triple-Negative Breast Cancer Stem Cells (TNBC CSCs). The Venn diagram illustrates the overlap between target genes related to SLC26A9 (left) and those associated with TNBC CSCs (right), identifying 457 shared genes through database retrieval and comparison. The accompanying bar and column graphs further visualize the distribution of specific and shared targets for both gene sets. These findings highlight the broad involvement and potential multi-pathway regulatory role of SLC26A9 within the TNBC CSC regulatory network, providing a molecular basis for subsequent PPI analyses and functional enrichment. The identification of 457 common genes forms the theoretical basis for systematically elucidating the “bridge” network hub role of SLC26A9 in TNBC CSCs and supports the development of multi-target therapies.

To comprehensively elucidate the molecular network by which SLC26A9 regulates TNBC CSCs, this study integrated multi-omics databases and network pharmacology tools. First, using the GeneCards database (https://www.genecards.org/Search), we systematically identified disease-associated targets with the keywords “SLC26A9” and “triple-negative breast cancer stem cells.” The target genes associated with SLC26A9 and those related to TNBC CSCs were then input into the online Venny 2.1 mapping platform (https://www.bioinformatics.com.cn/), revealing 457 overlapping target genes (Figure 1). These shared genes reflect the broad involvement of SLC26A9 in CSC regulatory networks and provide a foundation for subsequent protein–protein interaction (PPI) and functional enrichment analyses.

**FIGURE 2:**
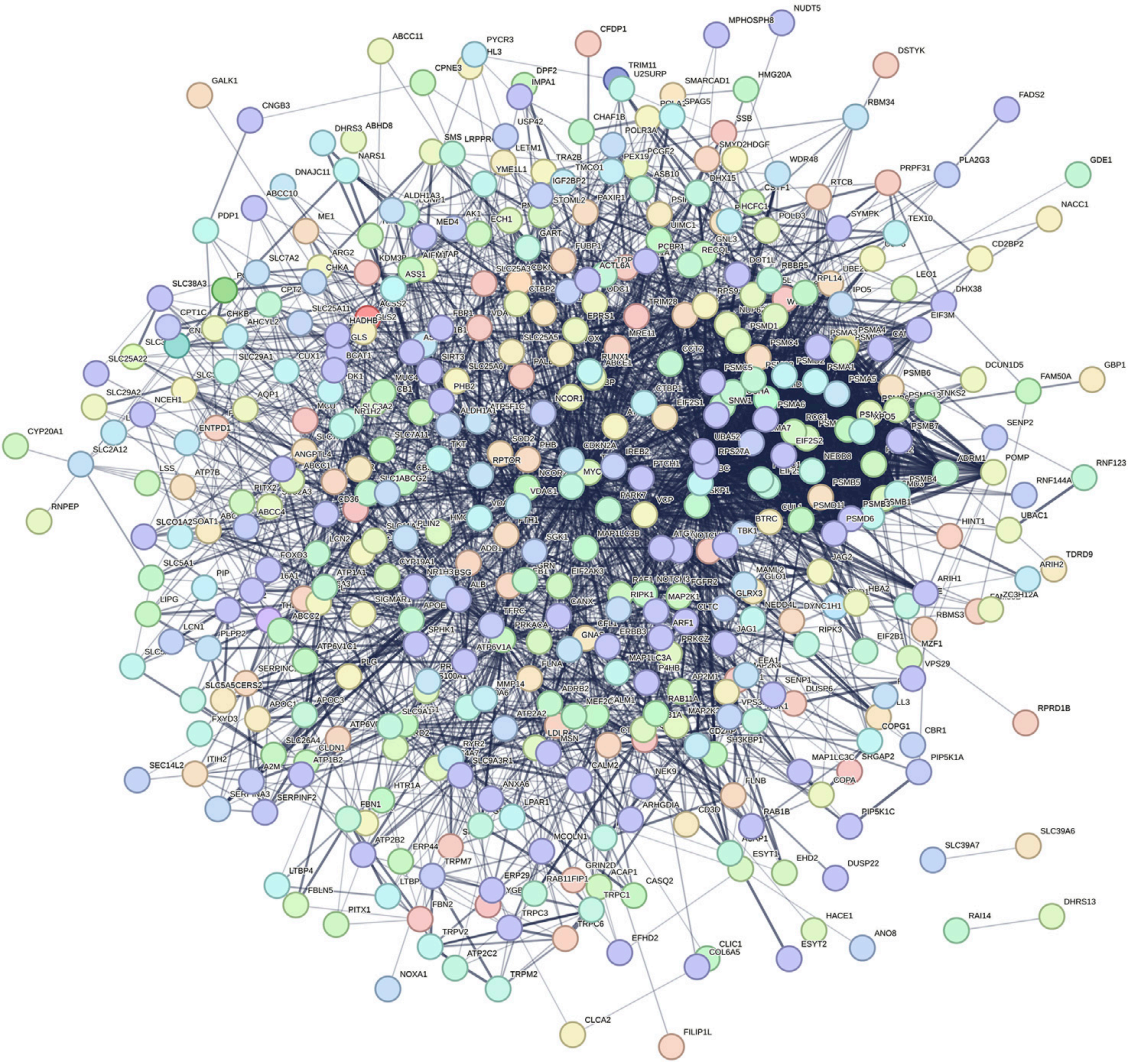
Protein–protein interaction (PPI) network of the 457 shared genes between SLC26A9 and TNBC CSCs. The network (STRING, *Homo sapiens*, combined score >0.4; unconnected nodes removed) is visualized in Cytoscape 3.9.1. Nodes represent proteins; edges represent predicted functional associations; node color intensity reflects degree centrality. TP53, MYC, and UBC emerge as high-degree nodes, suggesting candidate hub behavior based on topology. These observations are computational and hypothesis-generating, warranting functional validation.

The 457 overlapping target genes were uploaded to the STRING database (https://cn.string-db.org/), with the species set to “*Homo sapiens*” and a combined score threshold >0.4. Unrelated proteins were excluded to generate a refined PPI network of therapeutic targets (Figure 2). As shown in Figure 2, the PPI network visualization highlights TP53, MYC, and UBC as candidate high-centrality nodes within the shared gene set, suggesting potential multi-pathway coordination.

### 4.3 Protein–protein interaction (PPI) network analysis

**FIGURE 3:**
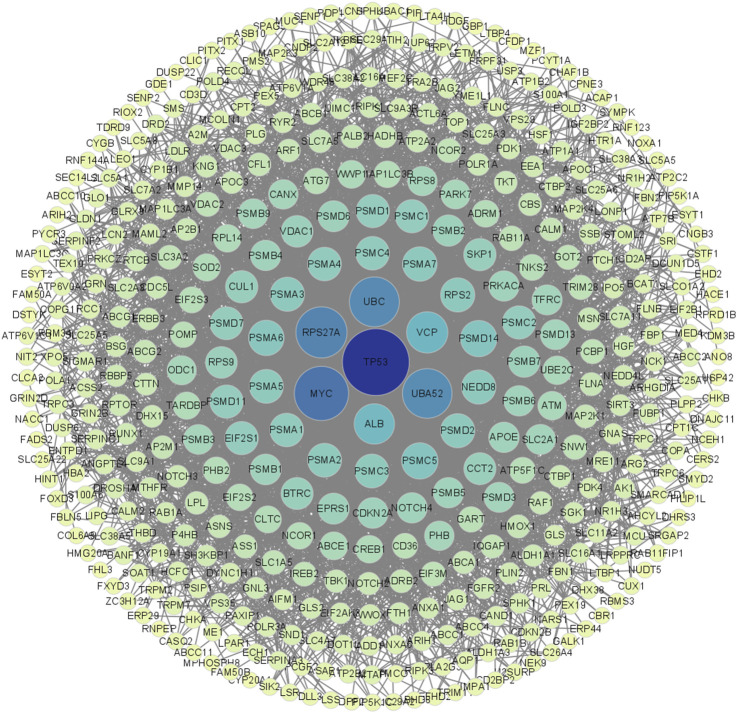
Visualization and Centrality Analysis of the Protein–Protein Interaction (PPI) Network of Shared Genes between SLC26A9 and Triple-Negative Breast Cancer Stem Cells (TNBC CSCs). The PPI network of the 457 overlapping genes was visualized using Cytoscape. Node color intensity represents centrality, with darker nodes indicating network hub proteins. The results show that key molecules such as TP53, UBC, MYC, and ALB occupy central positions within the network, forming a multi-layered concentric structure with high connectivity. TP53 is identified as the primary central node, suggesting that SLC26A9 may achieve multidimensional and precise regulation of CSC fate, drug resistance, and microenvironmental adaptation through interactions with core regulatory proteins like TP53. This figure visually illustrates the systems pharmacology regulatory network involving SLC26A9, TP53, and multiple cooperating proteins, providing a solid theoretical foundation for multi-target drug development.

The network was visualized using Cytoscape 3.9.1 (Figure 3). The results showed that TP53 occupies a central hub position within the network, suggesting that SLC26A9 may exert multidimensional regulation of CSC fate, drug resistance, and microenvironmental adaptation through TP53 and other core molecules. This “bridge-hub” network architecture highlights the potential systems pharmacology value of SLC26A9. It should be noted, however, that the current network analysis is primarily based on database integration, and actual molecular interaction networks in patient tissues may exhibit heterogeneity due to individual differences and gene mutations. Therefore, these conclusions require further validation through large-scale studies and functional experiments.

### 4.4 GO and KEGG functional enrichment analyses reveal the anticancer mechanisms of SLC26A9

**FIGURE 4:**
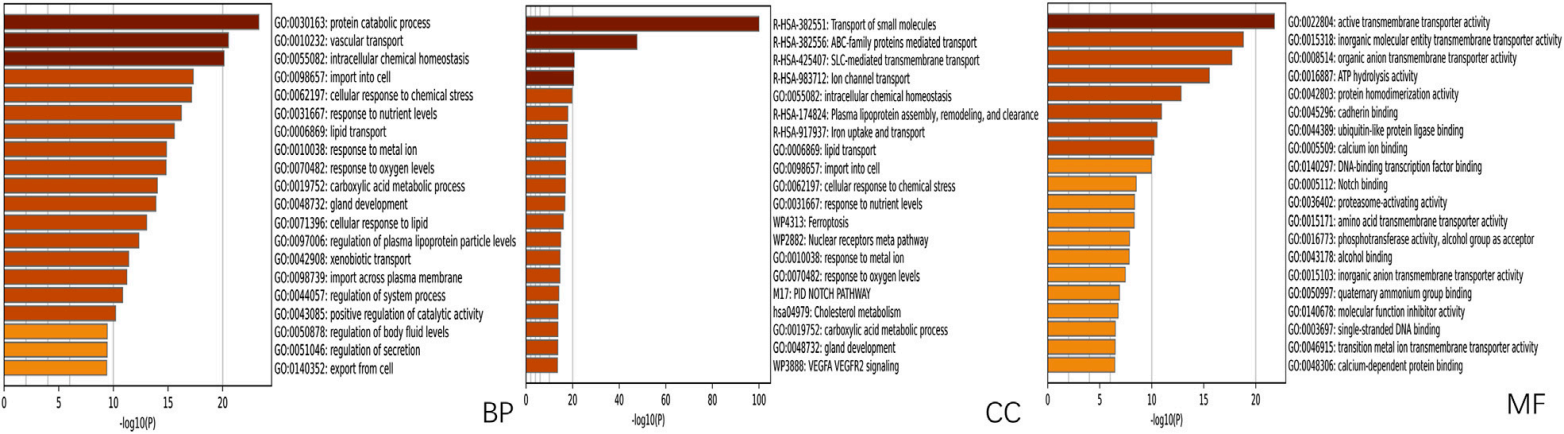
GO Functional Enrichment Analysis of Shared Genes between SLC26A9 and Triple-Negative Breast Cancer Stem Cells (TNBC CSCs). Gene Ontology (GO) enrichment analysis of the 457 overlapping SLC26A9–TNBC CSC genes was performed using the Metascape platform, highlighting major terms within the categories of biological process (BP), cellular component (CC), and molecular function (MF). The results show significant enrichment in protein metabolism, intracellular homeostasis, stress responses, organelle composition, and transmembrane transport, with prominent molecular functions in transporter activity, ligand binding, and enzyme regulation. These enriched pathways suggest that SLC26A9 regulates TNBC CSC self-renewal, drug resistance, and microenvironmental adaptation through complex signaling, metabolic, and transport networks.

**FIGURE 5:**
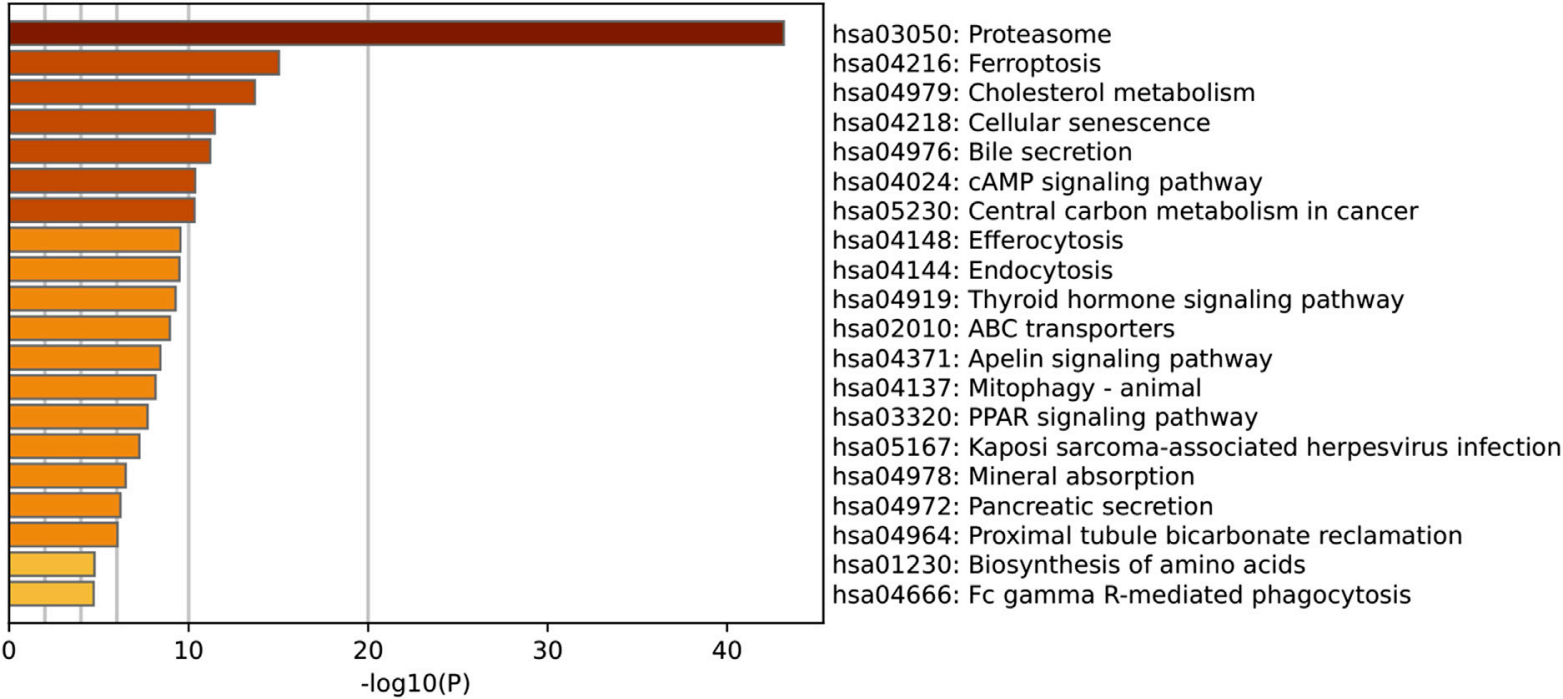
KEGG Pathway Enrichment Analysis of Shared Genes between SLC26A9 and Triple-Negative Breast Cancer Stem Cells (TNBC CSCs). KEGG pathway enrichment analysis was performed on the 457 overlapping SLC26A9–TNBC CSC genes. The figure displays the top 20 most significantly enriched pathways. The results indicate that these genes are prominently involved in the regulation of key pathways such as the proteasome, ferroptosis, cholesterol metabolism, cellular senescence, cAMP signaling, central carbon metabolism, and ABC transporters. These core pathways are closely linked to tumor cell self-renewal, drug resistance, and microenvironmental adaptation, further reinforcing the critical role of SLC26A9 as a multi-target “bridge” within the complex regulatory network of TNBC CSCs.

Using network pharmacology, we performed Gene Ontology (GO) functional analysis and Kyoto Encyclopedia of Genes and Genomes (KEGG) pathway enrichment analysis to elucidate the regulatory network involving SLC26A9 and its interacting proteins in TNBC stem cells. The shared target genes of “SLC26A9” and “triple-negative breast cancer stem cells” were input into the Metascape platform (https://metascape.org/gp/index.html#/main/step1), with “*Homo sapiens*” selected as the species. We further analyzed the biological processes (BP), cellular components (CC), and molecular functions (MF) GO terms, as well as signaling pathways associated with SLC26A9-mediated TNBC CSC regulation (Figures 4, 5).

The GO analysis results highlight that SLC26A9-related genes are significantly involved in several key biological processes, including protein metabolic processes, lipid transport, cellular responses to chemical stress, and responses to nutrient levels (Figure 4). Notably, SLC26A9 is associated with pathways such as iron uptake/transport and vascular transport. Among the enriched terms, the Notch signaling pathway emerges as a key axis that connects SLC26A9 with stemness maintenance and tumor progression. These pathways are crucial for regulating the self-renewal, drug resistance, and microenvironmental adaptation of TNBC CSCs. The results also indicate a strong enrichment of genes involved in cellular transport activities and transmembrane transporter protein functions, emphasizing the importance of metabolic and transport processes in TNBC pathogenesis.

These findings support the role of SLC26A9 in regulating TNBC CSCs through multi-pathway and multi-target interactions, including the Notch signaling pathway and metabolic adaptation mechanisms. These enriched terms provide a solid theoretical foundation for further research and offer guidance for designing multi-target therapies aimed at improving TNBC treatment outcomes. Overall, the enriched GO terms reveal coupling between stemness and metabolic/TME adaptation, suggesting that SLC26A9 plays a crucial role in TNBC stem cells and providing new insights for the development of novel multi-target drugs for TNBC treatment.

In addition, KEGG pathway enrichment analysis further clarified the potential biological mechanisms underlying the role of SLC26A9 in TNBC CSCs (Figure 5). The top 20 enriched pathways included proteasome, ferroptosis, cholesterol metabolism, cellular senescence, cyclic adenosine monophosphate (cAMP) signaling, and ABC transporters, all of which are closely related to tumor cell survival, metabolic reprogramming, and drug resistance. Among these, the ferroptosis and cholesterol metabolism pathways are particularly noteworthy, as both have been increasingly recognized as critical regulators of cancer stem cell plasticity and therapy resistance. The enrichment of proteasome and cellular senescence pathways suggests that SLC26A9 and its interacting partners may participate in protein homeostasis and stress adaptation within CSCs, whereas ABC transporter pathways highlight potential links to multidrug resistance mechanisms. Collectively, these KEGG results complement the GO findings by emphasizing that SLC26A9 functions as a bridge between ion transport, metabolic regulation, and signal transduction, thereby providing a systems-level explanation for its hypothesized role in maintaining CSC stemness and adaptability.

## 5 Structural basis of molecular modeling and multi-target drug development

Building on systems pharmacology and network pharmacology, structural biology and molecular modeling provide critical support for elucidating the mechanisms of SLC26A9 and its interacting proteins, as well as for validating the feasibility of multi-target drug design. To further clarify the precise regulatory mechanisms of the SLC26A9–TP53 signaling axis in TNBC CSCs, this study selected the small molecule S9-A13 (downloaded from PubChem, CID: 135986215) as a candidate compound. Detailed docking scores, binding residues, and interaction types are summarized in Table 1, providing complementary evidence for the binding characteristics of S9-A13. Molecular docking and molecular dynamics simulations were performed to systematically evaluate its binding characteristics and dynamic stability with SLC26A9 (Research Collaboratory for Structural Bioinformatics Protein Data Bank, RCSB PDB; PDB ID: 7CH1) and TP53 (RCSB PDB; PDB ID: 1TSR).

**TABLE 1:** Summary of key parameters for molecular docking and molecular dynamics.

Category	Item	Setting/version
Structures and ligand	Receptors	SLC26A9: PDB 7CH1; TP53: PDB 1TSR
	Ligand source	S9-A13 (PubChem CID: 135986215)
Docking	Preparation	MGLTools 1.5.7 (protonation, PDBQT conversion)
	Software and mode	AutoDock Vina 1.1.2; global docking
	Key parameters	Exhaustiveness = 16; num_modes = 30
MD platform	Software	GROMACS 2024.03
	Force fields/parameters	Protein: AMBER14SB; ligand: ACPYPE/GAFF
Solvent and box	Water model/box	TIP3P; octahedral periodic boundary conditions
	Ions and salinity	Na^+^/Cl^−^ to 0.15 mol/L, system neutralization
Energy minimization	Algorithm and steps	Steepest-descent, 50,000 steps
Equilibration	NVT	100 ps, V-rescale thermostat
	NPT	100 ps, Parrinello–Rahman barostat
Production MD	Conditions and timestep	100 ns; 300 K; 1 bar; 2 fs timestep
	Electrostatics/output	PME for long-range electrostatics; 1 frame/10 ps
Analyses	Trajectory	RMSD, RMSF, Rg, secondary-structure evolution, PCA/FEL, H-bonds
Free energy	MM/GBSA	gmx_MMPBSA on 90–100 ns stable window

Recent studies have confirmed that S9-A13 specifically inhibits SLC26A9-mediated anion exchange activity at the nanomolar level (half-maximal inhibitory concentration, IC50 = 90.9 ± 13.4 nM) without significant inhibition of other ion channels. This high specificity supports the potential of S9-A13 for precision drug design (Kunzelmann et al., 2023).

### 5.1 Molecular docking reveals a multi-target mechanism of action

Molecular docking simulations suggest that the candidate compound S9-A13 may exhibit a strong binding affinity to two key regulatory proteins, SLC26A9 and TP53. For SLC26A9, S9-A13 is predicted to form a stable interaction by binding to a specific site on the protein. Similarly, S9-A13 is computationally predicted to bind to a potential pocket on the tumor suppressor protein TP53, forming a putative stable complex.

These *in silico* findings should be regarded as hypothesis-generating rather than confirmatory. They indicate that S9-A13 could potentially interact with both SLC26A9 and TP53, suggesting a possible multi-target regulatory mechanism affecting key signaling pathways and the tumor microenvironment of cancer stem cells. However, as these results are derived entirely from computational modeling, they require further validation through biochemical binding assays and functional experiments.

**FIGURE 6:**
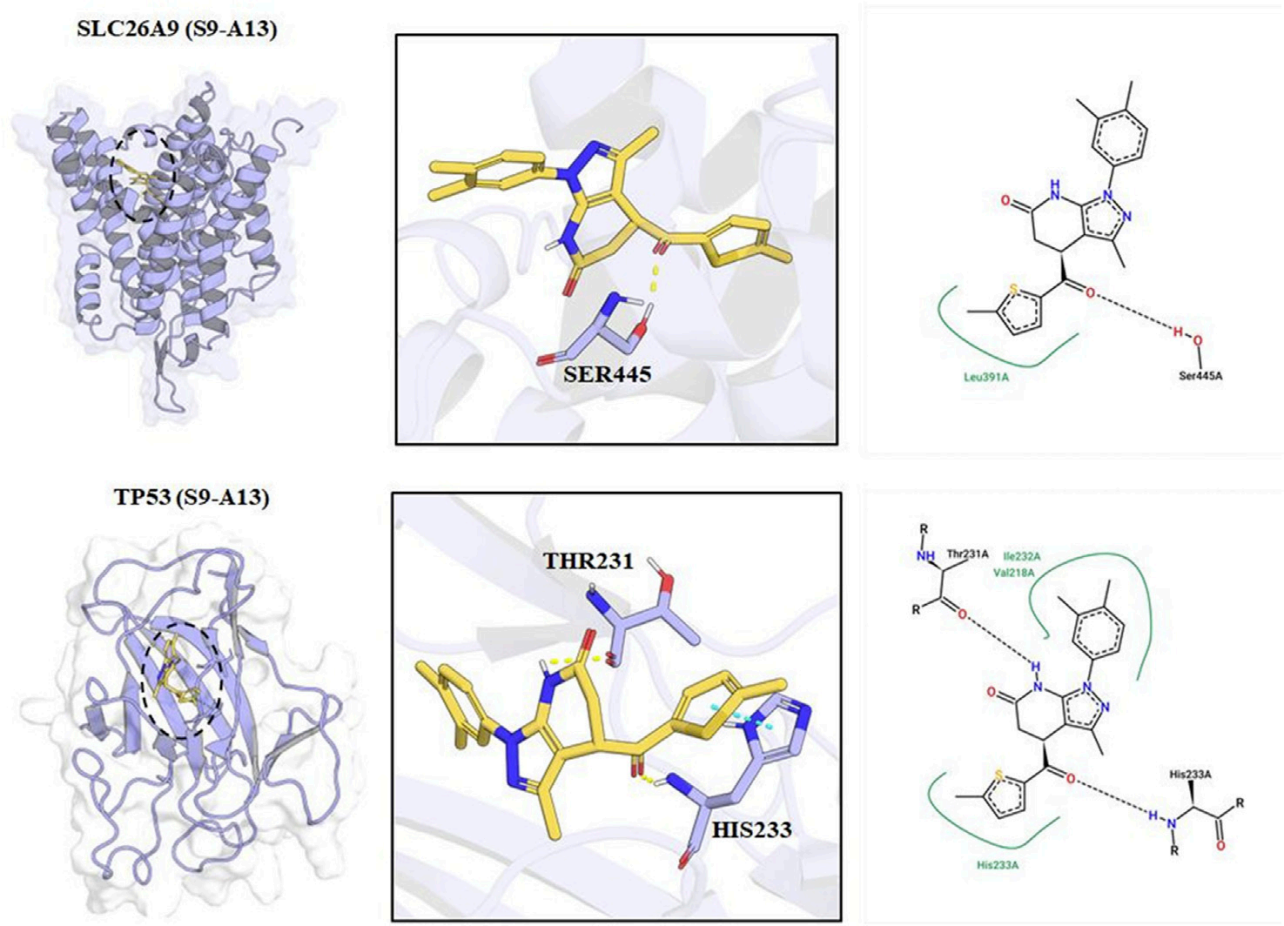
Molecular Docking Models and Key Interaction Sites of S9-A13 with SLC26A9 and TP53 Proteins. The left panels show the three-dimensional binding conformations of S9-A13 with SLC26A9 (top) and TP53 (bottom). The central panels provide close-up views, highlighting hydrogen bonds and hydrophobic interactions between S9-A13 and key residues—LEU391 and SER445 of SLC26A9, and THR231 and HIS233 of TP53. The right panels present schematic 2D interaction diagrams. Molecular dynamics simulations revealed that both complexes remained conformationally stable over 100 ns, with van der Waals interactions as the primary driving force. The calculated binding free energies were −34.47 kcal/mol for SLC26A9 and -25.65 kcal/mol for TP53, further supporting the high-affinity, multi-target binding properties of S9-A13.

Therefore, the interpretation that TP53 acts as a “central hub” within the SLC26A9-related network should be considered a theoretical inference pending experimental confirmation. This dual-target hypothesis provides a conceptual—rather than conclusive—foundation for the future development of precision therapeutic agents targeting TNBC stem cells (Figure 6).

### 5.2 Molecular dynamics simulations validate binding stability

Molecular dynamics simulations further demonstrated stable complex formation throughout 100 ns without significant dissociation or structural loosening.

**FIGURE 7:**
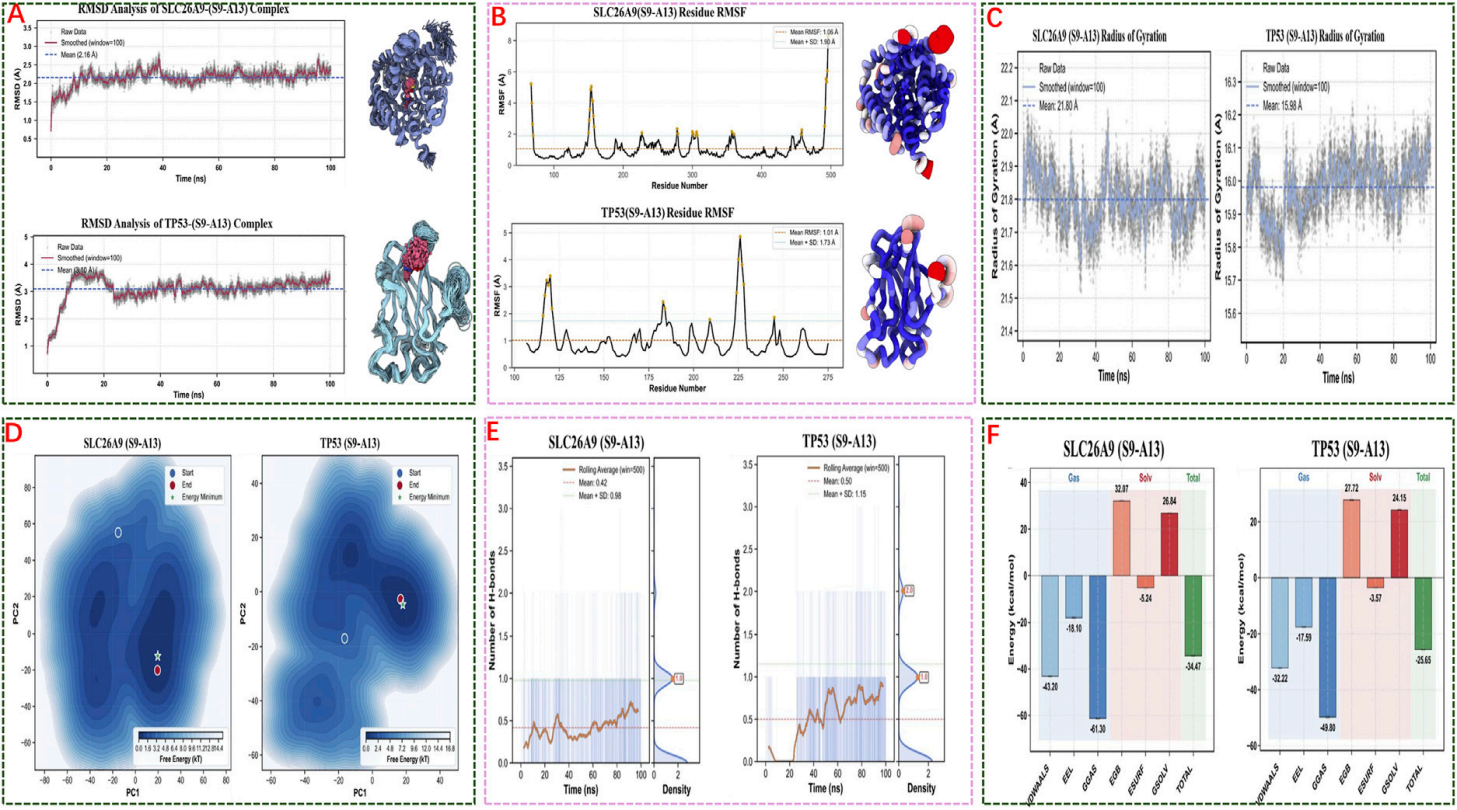
Molecular Dynamics Simulations and Energy Analysis of S9-A13 Complexes with SLC26A9 and TP53. **(A)** RMSD analysis shows that both S9-A13/SLC26A9 and S9-A13/TP53 complexes achieved structural stability over 100 ns of simulation, with no significant drift observed; **(B)** RMSF analysis indicates low overall residue fluctuation, with flexibility primarily localized to the protein termini, while the core structures remained stable; **(C)** Radius of gyration (Rg) measurements confirm that both complexes maintained a compact structure without abnormal expansion or collapse; **(D)** Free energy landscape (FEL) analysis based on principal component analysis (PCA) reveals convergence to low-energy basins during the latter stages of simulation, indicating thermodynamic stability; **(E)** Hydrogen bond analysis shows that binding of S9-A13 to both proteins is mainly driven by hydrophobic and van der Waals interactions, with limited and highly variable hydrogen bonding; **(F)** MM/GBSA energy decomposition indicates binding free energies of −34.47 kcal/mol (SLC26A9) and −25.65 kcal/mol (TP53), with spontaneous binding primarily driven by van der Waals and electrostatic interactions. Collectively, these results demonstrate the high-affinity, stable, multi-target binding of S9-A13 to SLC26A9 and TP53, providing an energetic and structural basis for systematic multi-target intervention in triple-negative breast cancer stem cells.

Detailed analyses indicated that binding of S9-A13 does not significantly alter the core structures of either protein, with only minor fluctuations detected in specific regions. Energy analysis further confirmed this stability, as the complexes reached progressively lower energy states over the course of the simulation, ultimately stabilizing at minimal energy levels (Figure 7).

Overall, these results suggest that the interactions between S9-A13 and both SLC26A9 and TP53 are stable and reliable, providing strong theoretical and experimental support for the potential of S9-A13 as a precision therapeutic targeting multiple key proteins to regulate cancer stem cell functions and the tumor microenvironment.

However, it should be noted that while molecular docking and dynamics simulations provide important theoretical insights into drug–target interactions, the simulated conditions differ from the true *in vivo* environment. Therefore, the efficacy and safety of small molecules such as S9-A13 must be further validated through comprehensive cellular, animal, and clinical studies. While supporting biophysical plausibility, MD/MM-GBSA results should be interpreted with caution due to differences from *in vivo* conditions; cellular and animal studies are needed to assess functional consequences and safety.

## 6 Discussion: structural insights and multi-target drug development

This work achieves a closed-loop validation from the network pharmacology level (“target–pathway–network”) to the molecular level (“structure–energy–dynamics”), demonstrating that S9-A13 can simultaneously act on network hubs such as SLC26A9 and TP53, enabling precise multi-target regulation across signaling axes and biological processes. These findings provide a robust theoretical foundation for optimizing multi-target drug development and combination strategies for TNBC CSCs, while also establishing a novel framework for translating systems pharmacology research into clinical applications.

Additionally, existing studies have validated the role of SLC26A9 in different types of cancer cells through *in vitro* experiments. For example, a study by Liu et al. (2022) demonstrated that the deletion of SLC26A9 in gastric cancer cells significantly inhibited cell proliferation and migration (Liu et al., 2022). Furthermore, a study by Ma et al. (2025) showcased the role of SLC26A9 in breast cancer, indicating that it promotes tumor cell proliferation and metastasis through the PI3K/AKT signaling pathway (Ma et al., 2025). These findings are consistent with our computational predictions and provide convergent support for the proposed mechanism. Building on these discoveries, we plan to further combine *in vitro* experiments, particularly using clustered regularly interspaced short palindromic repeats (CRISPR)/CRISPR-associated protein 9 (Cas9) technology to knock out the SLC26A9 gene, to explore its specific role in TNBC stem cells.

## 7 Conclusion and future perspectives

This review centers on triple-negative breast cancer stem cells (TNBC CSCs), systematically summarizing the full-spectrum regulatory mechanisms of SLC26A9 and its interacting proteins in CSC signaling networks, metabolic regulation, and tumor microenvironmental adaptation. Through integrated multi-omics database analysis, network pharmacology, and protein–protein interaction network studies, we propose for the first time that SLC26A9 acts as a “bridge” multi-target hub in the regulation of CSC self-renewal, drug resistance, and microenvironmental adaptation—particularly through its coordinated regulation with the TP53 signaling axis—offering a new molecular entry point for precise CSC intervention.

Building on this, our study combined molecular docking and molecular dynamics simulations to validate, at both the structural and energetic levels, the high-affinity, stable multi-target binding of the small molecule S9-A13 to SLC26A9 and TP53. This supports the rational design of agents that concomitantly target multiple pathways and networks in CSCs. The “systems pharmacology–structural simulation” closed-loop paradigm not only deepens theoretical understanding of the SLC26A9–TP53–CSC signaling axis in cancer stem cell regulation, but also serves as a valuable research model for the discovery and optimization of novel anti-TNBC CSC multi-target agents.

It is important to note that as an innovative therapeutic target, SLC26A9’s expression profile in normal tissues, potential side effects, and the specificity and safety of its targeting agents require further investigation. Future challenges include preclinical efficacy and safety assessment of multi-target drugs, optimization of delivery systems, and the application of real-world data to achieve clinical translation.

Looking forward, several research directions are recommended: dynamic multi-omics monitoring; drug delivery and combination strategies; artificial intelligence (AI) and high-throughput screening; and preclinical and translational research. Strengthen *in vitro* and *in vivo* functional evaluation and safety assessment of SLC26A9 and its targeting compounds, driving the clinical translation of candidate drugs for precision TNBC therapy.

In summary, the systematic investigation of SLC26A9 and its interacting proteins, along with multi-target drug development, offers new opportunities to overcome the challenges of TNBC CSC drug resistance and recurrence. This will help advance systems pharmacology and precision medicine, and may lead the way in the future of targeted therapies for cancer stem cells.

## Abbreviations

ABC: ATP-binding cassette; ALDH: aldehyde dehydrogenase; BP: biological process; CC: cellular component; CSC(s): cancer stem cell(s); ER: estrogen receptor; FEL: free energy landscape; GO: Gene Ontology; HER2: human epidermal growth factor receptor 2; IC50: half-maximal inhibitory concentration; JAK: Janus kinase; KEGG: Kyoto Encyclopedia of Genes and Genomes; MF: molecular function; MM/GBSA: molecular mechanics/generalized Born surface area; PCA: principal component analysis; PI3K: phosphoinositide 3-kinase; PPI: protein–protein interaction; Rg: radius of gyration; RMSD: root-mean-square deviation; RMSF: root-mean-square fluctuation; SLC26A9: solute carrier family 26 member 9; STAT: signal transducer and activator of transcription; TME: tumor microenvironment.
